# Transient congenital hyperinsulinism and hemolytic disease of a newborn despite rhesus D prophylaxis: a case report

**DOI:** 10.1186/s13256-021-03167-9

**Published:** 2021-11-26

**Authors:** Sandra Simony Tornoe Riis, Marianne Hoerby Joergensen, Kristina Fruerlund Rasmussen, Steffen Husby, Jane Preuss Hasselby, Lise Borgwardt, Klaus Brusgaard, Christina Ringmann Fagerberg, Henrik Thybo Christesen

**Affiliations:** 1 Institute of Clinical Research, University of Southern Denmark, Odense, Denmark; 2 The Paediatric and Adolescent Clinic 4072, Copenhagen University Hospital, Rigshospitalet, Copenhagen, Denmark; 3 Department of Clinical Immunology, Odense University Hospital, Odense, Denmark; 4 Department of Pathology, Copenhagen University Hospital, Rigshospitalet, Copenhagen, Denmark; 5 Clinic for Clinical Physiology, Nuclear Medicine and PET, Copenhagen University Hospital, Rigshospitalet, Copenhagen, Denmark; 6 Department of Clinical Genetics, Odense University Hospital, Odense, Denmark; 7 Hans Christian Andersen Children’s Hospital, Odense University Hospital, Odense, Denmark

**Keywords:** Case report, Newborn, Rhesus immunization, Transient congenital hyperinsulinism, Conjugated hyperbilirubinemia

## Abstract

**Background:**

In neonates, rhesus D alloimmunization despite anti-D immunoglobulin prophylaxis is rare and often unexplained. Rhesus D alloimmunization can lead to hemolytic disease of the newborn with anemia and unconjugated hyperbilirubinemia. In past reports, transient congenital hyperinsulinism has been described as a rare complication of rhesus D alloimmunization. Our case report illustrates that rhesus D alloimmunization can result in a pseudosyndrome with severe congenital hyperinsulinism, anemia, and conjugated hyperbilirubinemia, despite correctly administered anti-D immunoglobulin prophylaxis.

**Case presentation:**

We report of a 36-year-old, Caucasian gravida 1, para 1 mother with A RhD negative blood type who received routine antenatal anti-D immunoglobulin prophylaxis. Her full term newborn boy presented with severe congenital hyperinsulinism, anemia, and conjugated hyperbilirubinemia up to 295 µmol/L (ref. < 9), accounting for 64% of the total bilirubin. Syndromic congenital hyperinsulinism was suspected. Examinations showed a positive direct antiglobulin test, initially interpreted as caused by irregular antibodies; diffuse congenital hyperinsulinism by 18F-DOPA positron emission tomography/computed tomography scan; normal genetic analyses for congenital hyperinsulinism; mildly elevated liver enzymes; delayed, but present bile excretion by Tc99m-hepatobiliary iminodiacetic acid scintigraphy; and cholestasis and mild fibrosis by liver biopsy. The maternal anti-D titer was 1:16,000 day 20 postpartum. Y-chromosome material in the mother’s blood could not be identified. This could, however, not exclude late intrapartum fetomaternal hemorrhage as the cause of immunization. No causative genetic findings were deetrmined by trio whole exome sequencing*.* The child went into clinical remission after 5.5 months.

**Conclusion:**

Our case demonstrates that rhesus D alloimmunization may present as a pseudosyndrome with transient congenital hyperinsulinism, anemia, and inspissated bile syndrome with conjugated hyperbilirubinaemia, despite anti-D immunoglobulin prophylaxis, possibly due to late fetomaternal hemorrhage.

## Background

Congenital hyperinsulinism (CHI) is characterized by hyperinsulinemic hypoglycemia, which can be transient, intermittent, or persistent. Transient CHI (tCHI) commonly occurs in newborns with an underlying genetic defect or maternal history of diabetes, but is also associated with perinatal stress conditions such as intrauterine growth restriction, birth asphyxia, and maternal preeclampsia [1]. A rare cause of tCHI is hemolytic disease of the newborn (HDN) due to severe rhesus D (RhD) alloimmunization. However, no cases have been described since the late 1960s, probably due to the introduction of anti-RhD immunoglobulin (anti-D Ig) for at-risk RhD negative women [2].

Conjugated hyperbilirubinemia (CHB) in the newborn can be caused by infections, metabolic, genetic, syndromic, biliary, cardiovascular, or endocrine conditions, whereas the hemolysis due to HDN usually results in unconjugated hyperbilirubinemia [3]. Although the combination of tCHI and HDN is known, the combination of tCHI and CHB has only been described in patients with mutations in the hepatocyte nuclear factor-4 alpha (*HNF4A*) and in whole-genomic, mosaic paternal uniparental disomy (including Beckwith–Wiedemann syndrome) [4, 5]. We present a novel, pseudosyndromatic combination of severe tCHI, anemia, and severe CHB owing to RhD alloimmunization despite antenatal anti-D Ig prophylaxis.

## Case presentation

The male patient was born vaginally at a first-level hospital after 38 weeks gestation to a 36-year-old, Caucasian and non-obese gravida 1 para 1 mother with A RhD negative blood type. The standardized Danish screening and prenatal care guidelines were followed showing normal fetal ultrasound scans at 13 and 20 weeks, blood type antibody screenings at 13 and 25 weeks, and fetal blood type A RhD positive. That led to routine prophylaxis with 300 µg anti-D Ig to the mother at 30 weeks and shortly after birth. As no indications of increased risk of immunization were identified, no further investigations were performed during the pregnancy, which was otherwise uncomplicated without appreciable intrapartum fetomaternal hemorrhage (FMH). Shortly after birth, the mother developed transient hypertension with blood pressure up to 160/90 mmHg and pronounced edema for 4–5 days. Proteinuria was not assessed, but postpartum preeclampsia was retrospectively suggested.

**Fig. 1:**
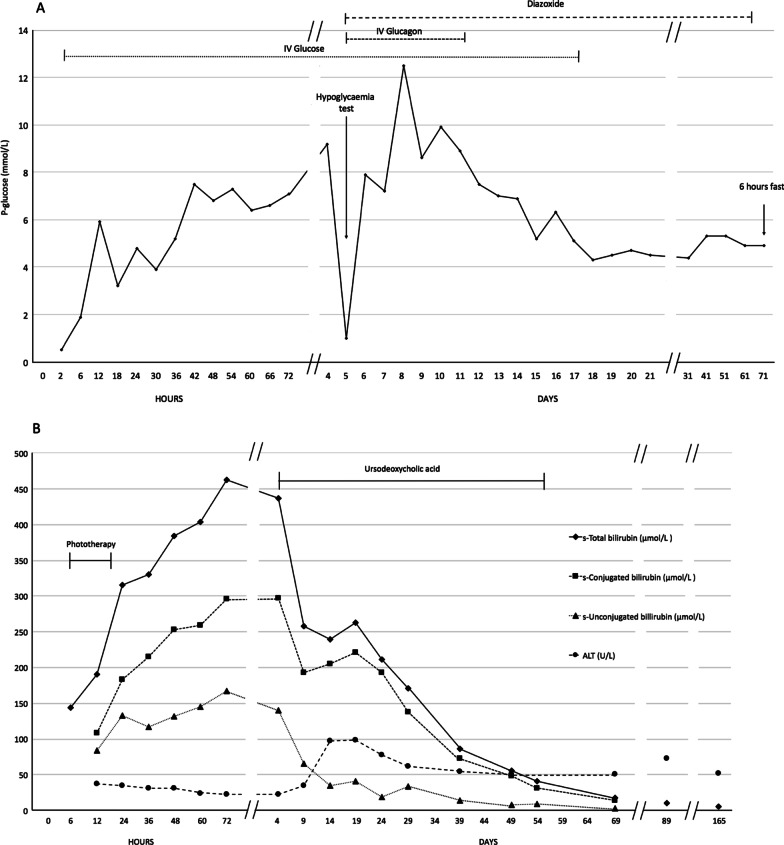
Timeline of glucose and bilirubin levels associated with hemolytic disease of the newborn. **A** P-glucose levels and treatment with IV glucose, IV glucagon, and diazoxide. **B** Total s-bilirubin, s-conjugated bilirubin, s-unconjugated bilirubin, and ALT levels, and treatment with phototherapy and ursodeoxycholic acid.

The boy was born vaginally at a first-level hospital, with a birth weight of 3080 g, cord pH 7.3, and Apgar score 10. No dysmorphic features were noted. He desaturated to 24% by pulse oximetry shortly after birth and received nasal continuous positive airway pressure with 100% FiO_2_ for 6 hours. At 2 hours of age, plasma glucose was 0.5 mmol/L (ref. > 2.5 mmol/L) and hemoglobin was 4.5 mmol/L (ref. 9.4–14.4 mmol/L). He was promptly treated with umbilical vein glucose infusion and a blood transfusion (Fig. 1A). No cranial ultrasound scan was performed. At 6 hours of age he developed jaundice with serum bilirubin of 144 µmol/L and phototherapy was initiated (Fig. 1B), after which he was transferred to the Copenhagen University Hospital for tertiary hospital level management. Here, a blood film showed leukoerythroblastosis and mild thrombocytopenia with no signs of leukemia.

On day 2, abdominal ultrasound identified hepatosplenomegaly, but a normal gall bladder. A direct antiglobulin test (DAT) was strongly positive. An irregular blood type alloimmunization was suspected, supported by lactate dehydrogenase level of 66 µkat/L (ref. 2.17–12.5 µkat/L), reticulocyte count 246 × 10^9^/L (ref. 79–222 × 10^9^/L), ferritin 528 µg/L (ref. 10–400 µg/L), haptoglobin < 0.1 g/L, and lactate 6.3 mmol/L (ref. 0.5–2.5 mmol/L).

On day 4, total bilirubin reached 462 µmol/L, unconjugated bilirubin was 167 µmol/L, and conjugated bilirubin (CB) was 295 µmol/L (64% of total bilirubin), for which he started treatment with ursodeoxycholic acid 50 mg/kg. Liver enzyme tests including alanine aminotransferase (ALT) and gamma-glutamyl transferase (GGT) were normal, as was the international normalized ratio (INR), excluding acute liver failure.

A persistently high intravenous glucose demand up to 28 mg/kg/minutes led to a short hypoglycemia test on day 5. Plasma glucose fell to 1.0 mmol/L with a simultaneous insulin measurement of 51 pmol/L (ref. 10–125 pmol/L) and C-peptide of 1870 pmol/L(ref. 379–1630 pmol/L) compatible with hyperinsulinism. He began treatment with diazoxide 10 mg/kg/day and glucagon 2.5 µg/kg/hour, which reduced but did not eliminate the need for intravenous glucose. Dried blood spot and urine metabolic screening, plasma amino acids, bicarbonate, ammonia, ketone levels, pituitary, and thyroid hormones were normal.

**Fig. 2:**
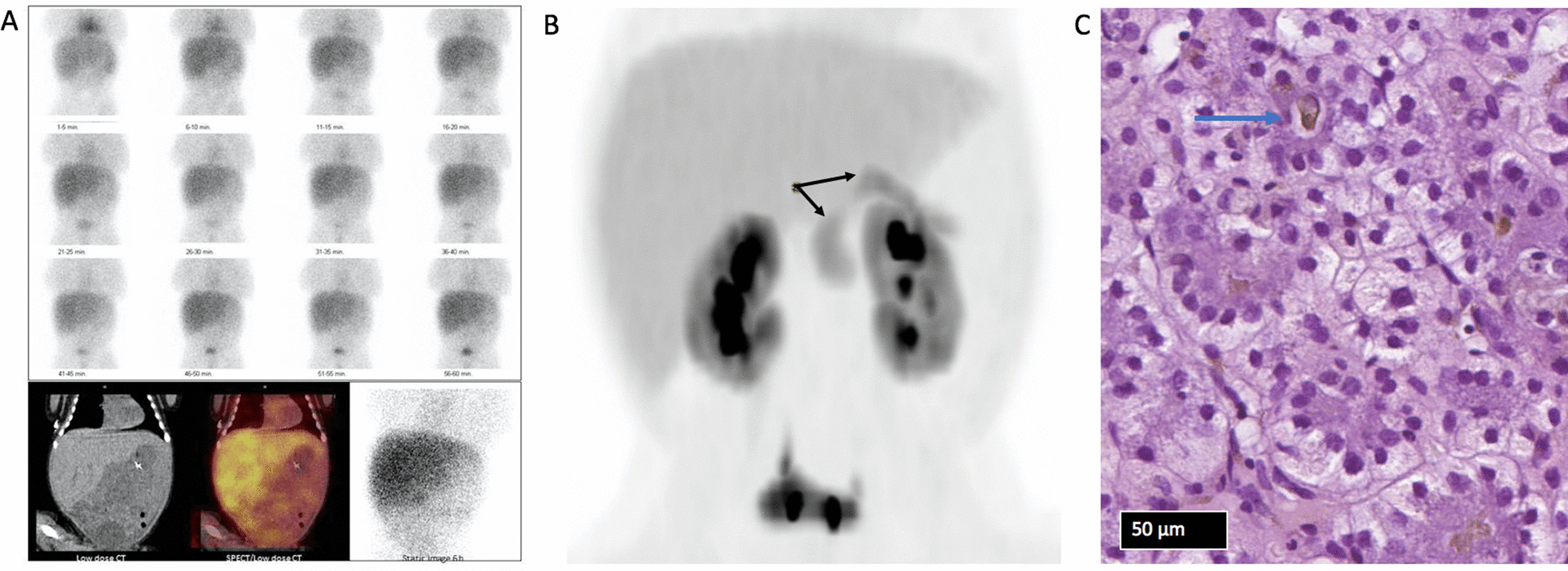
Diagnostic imaging of the liver, bile ducts, and pancreas, and a liver biopsy. **A** A dynamic scintigraphy at 1 hour was performed, supplemented with static images after 3, 6, and 24 hours. After 6 hours the static image was supplemented with SPECT/low dose CT. It showed delayed uptake in the liver parenchyma, delayed clearance of the blood pool and the liver parenchyma, and delayed and sparse distribution of the gallbladder to the intestines. SPECT/low dose CT was performed after 6 hours to verify the sparse uptake in the intestines. **B** 18F-DOPA PET/CT with diffuse uptake of DOPA throughout the entire pancreas. Black arrows demonstrating the pancreas. **C** Hematoxylin and eosin stained slide of the liver biopsy revealed hepatocytic rosette formation and marked cholestasis with bile plugs. Blue arrow indicating a bile plug

A Tc99m-hepatobiliary iminodiacetic acid scintigraphy with single photon emission computed tomography (SPECT)/low dose computed tomography (CT) on day 6 showed very delayed but intact bile excretion, excluding gall duct atresia and severe scintigraphic malfunction of the hepatocytes (Fig. 2A). Repeat liver enzymes tests were mostly normal except ALT, which increased from day 9 (Fig. 1B). Routine workup for congenital liver disease including alpha-1-antitrypsin, creatinine kinase, immunoglobulins, herpes simplex virus, toxoplasmosis, cytomegalovirus, parvovirus B19, and hepatitis B tests all returned normal. Ferritin was normal at admission and became only slightly elevated, peaking at 3450 µg/L, thus gestational alloimmune liver disease was not suspected.

Evaluation for CHI included an 18F-DOPA PET/CT scan on day 13 showed diffuse labeling of the pancreas, Fig. 2B. A next generation sequencing (NGS) panel for 11 genes in CHI including *ABCC8*, *KCNJ11*, *GCK*, *GLUD1*, *HADH*, *HNF1A*, *HNF4A*, *INS*, *INSR*, *SLC16A1*, and *UCP2* and a whole exome sequencing panel with > 2000 genes for diseases in childhood were all normal. This panel also included a cholestatic panel. Intravenous glucose was discontinued on day 17, but severe elevated CB continued (up to 84% of s-bilirubin). Magnetic resonance cholangiopancreatography on day 23 was normal apart from focal steatosis.

The longstanding anemia led to a diagnosis of RhD alloimmunization on day 20. The mother at that time had a strongly positive anti-D titer of 1:16,000 and the son had a titee of 1:256. A trio whole exome sequencing revealed no definite genetic cause of the anti-D prophylaxis failure. A heterozygous, maternal DNA variant in the complement regulatory protein gene *CD46* was identified on day 52: *c.1148C>T, p.Thr.383IIe*. This variant has not previously been functionally classified, except in relation to atypical hemolytic uremic syndrome. As *CD46* mutations may cause complement pathway abnormalities, an examination of the functional capacity in the classic, alternative, and lectin complement pathway was performed on day 90 in the mother with normal maternal results. In the child, the functional capacity of the classical and alternative pathway was reduced compared to reference values in adults, 42% (ref. 61–162%) and 58 % (ref. 64–130%), respectively.

In search for other causes for late alloimmunisation, foetal microchimerism was investigated by real time PCR, Y-chromosome material in the mother’s blood could not be identified. This could, however, not exclude late intrapartum foetomaternal haemorrhage. A search for other patients in Denmark and Europe with RhD alloimmunisation despite anti-D Ig was performed. The manufacturer had not registered similar cases in the organisation with the same or other batch numbers, so production errors of anti-D Ig were considered highly unlikely.

A liver biopsy was prompted by a rise in INR to 1.9 (ref. 0.9–1.6) on day 31 and phytomenadione was administered. The biopsy showed cholestasis with hepatocytic rosette formation, bile plugs and copper associated protein, and iron deposits and mild perisinusoidal fibrosis, but no ductopenia, steatosis, or periodic acid-Schiff (PAS) positive globules, thus unspecific changes with no evidence of metabolic disease, Allagille syndrome, or biliary atresia (Fig. 2C). Retrospectively, the biopsy was compatible with inspissated bile syndrome.

INR and CHB gradually normalized and ursodeoxycholic was discontinued on day 55. Diazoxide and nasogastric tube feeding were discontinued on day 61. Clinical remission was attained at the age of 5.5 months with normal eating pattern, weight gain, and clinical examination; normal spleen and liver size by abdominal ultrasound with absence of arterial hyperplasia of the hepatic artery and normal flow in the portal vein; normal blood cell counts (hemoglobin 7.5 mmol/L, leukocytes 10.1 × 10^9^/L with normal differential count, thrombocytes 405 × 10^9^/L), CB, INR, and ALT levels; and a 6-hour fasting test without hypoglycemia.

## Discussion

We report a combination of severe CHI and CHB caused by RhD alloimmunization despite antenatal anti-D Ig prophylaxis.

The RhD alloimmunization HDN was not expected when the newborn presented with significant anemia, because of the antenatal prophylaxis, the negative antibody screens and the absence of recognized immunizing events. Accordingly, no data on the severity and duration of fetal anemia were obtained. The rare possibility of HDN led to a delay in the diagnosis of RhD alloimmunization. Instead, the first positive DAT led to the presumption of an irregular blood type immunization, even though a broad antibody screening, which takes into account all the clinically relevant blood type antibodies, was performed early in the pregnancy. The co-occurrence of tCHI and CHB led to a fruitless search for a unifying syndrome.

Single-dose antenatal anti-D Ig administration in week 30 reduces the risk of RhD negative immunization in the mother from about 0.67% to 0.31%, compared with only postnatal anti-D Ig administration [6]. In general, the majority of immunizations are thought to be caused by occult or “silent” FMH. However our search for Y-chromosomes in the mother could not confirm this possibility. Our antibody screen in pregnancy at 25 weeks gestation is validated to detect IgG antibody activity down to 0.02 IU/mL, which is below the international limit of 0.05 IU/mL. Despite the negative screen result, we could not exclude a later upcoming event of immunization or the possibility that the mother was already immunized with a small amount of antibodies. Most plausible, however, is that the mother became immunized owing to fetomaternal hemorrhage in the period shortly before or between the antibody screen and the administration of anti-D Ig in week 30.

Severe CHB occurs in 3.5% of neonates with HDN after intrauterine blood transfusion, causing highly elevated ferritin [3]. Our patient had no intrauterine transfusions and did not present with severely increased ferritin levels. It is conceivable, however, that our patient may have developed CHB because of an excessive bilirubin load, caused by HDN owing to RhD alloimmunization in combination with hypoxia within the inspissated bile syndrome, as described in other cases reporting CHB as a complication of HDN [7]. The hemolytic anemia, mild thrombocytopenia, and splenomegaly had similarities with autoimmune lymphoproliferative syndrome (ALPS), but no further investigations into ALPS were performed because of the normal plasma immunoglobulins, normal exome sequencing, and clinical remission by age 5.5 months.

The CHI in our patient was transient with no identified genetic cause, rendering perinatal stress as the most likely cause of the hyperinsulinism. The pathophysiology of the beta cell function in several disorders with perinatal stress tCHI is not yet understood, although hyperlactatemia has been suggested as one cause, among others [8]. A previous report on tCHI in HDN suggested a direct effect of anti-RhD antibodies on the beta cells [9], but no other reports support the findings of RhD antigens expressed on islet cells, essential for a stimulating effect.

Our patient had both anemia and hypoxia with hyperlactatemia compatible with perinatal stress as the cause of tCHI. Indeed, hypoxia affects beta cell differentiation *in vitro*, and may result in putative mild, anabolic, and normoglycemic hyperinsulinism *in utero.* The transitional physiological hypoglycemia during the first days after birth may be driven by the lower oxygen supply in fetal life, in our case as a result of reduced hemoglobin concentration. Studies should be performed to investigate longstanding hypoxia as a common cause of physiological transient neonatal hypoglycemia and perinatal stress tCHI.

Our search for syndromes with both tCHI and CHB revealed a maternal *CD46* missense variant, recently reclassified from likely pathogenic to likely benign with respect to atypical hemolytic uremic syndrome. In general, heterozygous *CD46* polymorphisms are associated with both atypical hemolytic uremic syndrome and hemolytic uremic syndrome, but have also been linked to spontaneous miscarriages and to preeclampsia in patients with autoimmune diseases [10]. Dominant mutations in *CD46* may lead to dysregulation of the complement system with inexpedient activation of the alternative pathway and, hence, adverse immunological reactions. We speculated whether the *CD46* variant could be a novel cause of failure of anti-D Ig prophylaxis and potentially explain the postpartum signs of preeclampsia, which may have aggravated the perinatal stress tCHI. However, the functional capacity in the complement pathways was normal on day 90 in the mother and the lower values in the newborn may represent physiological immaturity of the complement system. Taken together, our data did not prove a role of *CD46* in the anti-D prophylaxis failure, but opens for further studies on this issue.

## Conclusion

RhD alloimmunization can present as severe perinatal stress tCHI, anemia, and severe CHB, despite anti-D prophylaxis.

## Data Availability

Not applicable.

## Abbreviations

CHI: Congenital hyperinsulinism
tCHI: Transient congenital hyperinsulinism
HDN: Hemolytic disease of the newborn
RhD: Rhesus D
anti-D-Ig: Anti-RhD immunoglobulin
CHB: Conjugated hyperbilirubinemia
